# Mechanism and Timescales of Reversible p‐Doping of Methylammonium Lead Triiodide by Oxygen

**DOI:** 10.1002/adma.202100211

**Published:** 2021-05-03

**Authors:** Dongguen Shin, Fengshuo Zu, Ayala V. Cohen, Yeonjin Yi, Leeor Kronik, Norbert Koch

**Affiliations:** ^1^ Institut für Physik & IRIS Adlershof Humboldt‐Universität zu Berlin 12489 Berlin Germany; ^2^ Helmholtz‐Zentrum Berlin für Materialien und Energie GmbH 12489 Berlin Germany; ^3^ Department of Molecular Chemistry and Materials Science Weizmann Institute of Science Rehovoth 76100 Israel; ^4^ Institute of Physics and Applied Physics & Van der Waals Materials Research Center Yonsei University Seoul 03722 Republic of Korea

**Keywords:** doping, energy level alignment, metal halide perovskites, oxygen

## Abstract

Understanding and controlling the energy level alignment at interfaces with metal halide perovskites (MHPs) is essential for realizing the full potential of these materials for use in optoelectronic devices. To date, however, the basic electronic properties of MHPs are still under debate. Particularly, reported Fermi level positions in the energy gap vary from indicating strong n‐ to strong p‐type character for nominally identical materials, raising serious questions about intrinsic and extrinsic defects as dopants. ​In this work, photoemission experiments demonstrate that thin films of the prototypical methylammonium lead triiodide (MAPbI_3_) behave like an intrinsic semiconductor in the absence of oxygen. Oxygen is then shown to be able to reversibly diffuse into and out of the MAPbI_3_ bulk, requiring rather long saturation timescales of ≈1 h (in: ambient air) and over 10 h (out: ultrahigh vacuum), for few 100 nm thick films. Oxygen in the bulk leads to pronounced p‐doping, positioning the Fermi level universally ≈0.55 eV above the valence band maximum. The key doping mechanism is suggested to be molecular oxygen substitution of iodine vacancies, supported by density functional theory calculations. This insight rationalizes previous and future electronic property studies of MHPs and calls for meticulous oxygen exposure protocols.

## Graphical abstract

Methylammonium lead triiodide thin films exhibit intrinsic semiconductor electronic properties in the absence of oxygen. Exposure to oxygen or air leads to pronounced p‐doping throughout the films, predominantly due to molecular oxygen substitution of iodine vacancies. This mechanism is reversible, and oxygen‐doped films return to intrinsic upon storage in ultrahigh vacuum.

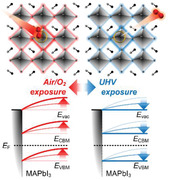

## 1 Introduction

Metal halide perovskites (MHPs) are promising optoelectronic materials because they can be solution‐fabricated at low cost and yet exhibit outstanding properties, e.g., high optical absorption coefficients, high charge carrier mobility, and long charge carrier diffusion length.^[^ ^1^, ^2^, ^3^, ^4^, ^5^ ^]^ These exceptional properties have led to a diverse scope of applications including solar cells, light‐emitting diodes, lasers, and photosensors.^[^ ^6^, ^7^, ^8^, ^9^, ^10^, ^11^ ^]^ MHP‐based solar cells, in particular, have exhibited a remarkably rapid increase in power conversion efficiency, which has now surpassed 25% in single‐junction cells and 28% in tandem cells.^[^ ^12^ ^]^

Despite this remarkable progress, fundamental questions regarding optoelectronic material and device properties of MHPs have not been fully addressed.^[^ ^13^ ^]^ One important issue, which is at the center of this work, is the strong variation of the Fermi level (*E*_F_) position in the energy gap of MHPs, with nominally undoped MHPs reported to exhibit behavior ranging from n‐type to p‐type.^[^ ^14^, ^15^, ^16^, ^17^, ^18^, ^19^, ^20^ ^]^ For example, Schulz et al. showed that *E*_F_ in methylammonium lead triiodide (MAPbI_3_) can be shifted by as much as 0.7 eV solely by varying the substrate from TiO_2_ to NiO_x_.^[^ ^17^ ^]^ In contrast, Zohar et al. found that single‐cation‐based perovskites (e.g., MAPbBr_3_ and CsPbBr_3_) exhibited a constant *E*_F_ position independent of the substrate.^[^ ^18^ ^]^ Moreover, Olthof reported the relation between MHP *E*_F_ position and substrate work function to exhibit considerable scatter, in excess of 1 eV.^[^ ^21^ ^]^ Such conflicting observations have been tentatively ascribed to differences in film stoichiometry, sample preparation condition and method, and history of sample handling (e.g., air exposure).^[^ ^22^ ^]^ For example, it has been shown that the sample work function can be strongly influenced by the stoichiometric composition.^[^ ^20^, ^21^ ^]^ In addition, depending on the presence of surface states, surface band bending can further complicate the interpretation of MHP energy levels as a function of substrate.^[^ ^23^ ^]^ Finally, different environmental conditions, originating from sample preparation and/or handling, have been shown to lead to inconsistent behavior.^[^ ^24^, ^25^, ^26^, ^27^, ^28^, ^29^, ^30^, ^31^, ^32^ ^]^ Therefore, a thorough and systematic investigation of energy level alignment mechanisms at perovskite/substrate interfaces is highly needed.

Here, we address this issue by studying the dependence of the electronic properties of MAPbI_3_ thin films prepared in two different environments, i.e., N_2_ and air, on the substrate work function, *Φ*_sub_, using ultraviolet photoelectron spectroscopy (UPS). We find that *E*_F_ of MAPbI_3_ prepared in inert gas changes significantly from seemingly n‐type to p‐type, by as much as 0.84 eV, revealing a so‐called *Z*‐curve behavior,^[^ ^33^ ^]^ which is a characteristic of a very low intrinsic doping level and negligible surface state density. In contrast, the energy levels of air‐prepared MAPbI_3_ films exhibit a pronounced p‐type character, independent of *Φ*_sub_. We further find, from controlled oxygen and vacuum exposure experiments, that oxygen p‐dopes MAPbI_3_ in a manner that can be fully reverted by storage in vacuum. With the help of density functional theory (DFT), we suggest that the bulk p‐doping effect originates from oxygen molecules substituting native iodine vacancy sites, eventually aided by oxygen association to iodine interstitials. Oxygen also reduces metallic‐lead‐related surface states irreversibly and thus facilitates surface defect passivation.

## 2 Results and Discussion

### 2.1 Energy Level Alignment of MAPbI_3_ as a Function of Substrate Work Function

The binding energy of the valence band maximum (VBM) (*E*_VBM_) with respect to *E*_F_, and the work function (*Φ*) of MAPbI_3_ films, fabricated in N_2_ and air on a variety of substrates, as well as the work functions of all bare substrates were obtained from UPS measurements (raw UPS data for all MAPbI_3_/substrate and bare substrate samples are given in Figures S1–S3 of the Supporting Information). *E*_VBM_ values were extrapolated from logarithmic photoelectron intensity plots owing to the comparably low density of states at the top of the MAPbI_3_ valence band, as suggested by Endres et al.^[^ ^34^ ^]^ and confirmed later by angle‐resolved UPS data.^[^ ^35^ ^]^ Additional surface photovoltage measurements of the samples discussed here showed no energy level shifts for UV excitation intensities varied by a factor of ≈100, indicating a negligible surface state density and the absence of surface band bending in the ground state^[^ ^36^ ^]^ (see Figure S4 in the Supporting Information).

**Figure 1:**
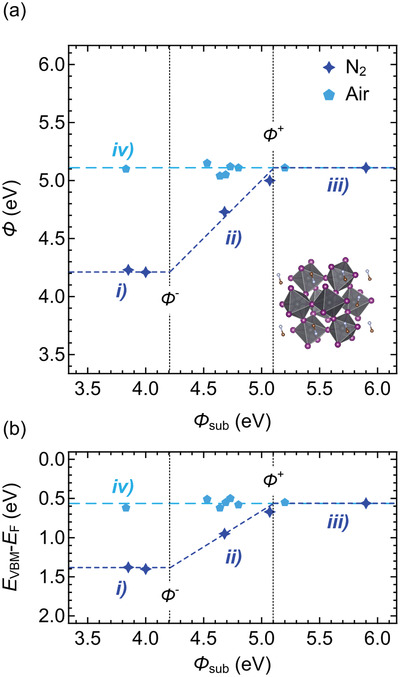
a) *Φ* values and b) valence band maxima (*E*_VBM_ − *E*_F_) of MAPbI_3_ films prepared in N_2_ and air, as a function of substrate work function (*Φ*_sub_). The dashed lines are guides to the eye. For samples prepared in N_2_, region (i) corresponds to the *E*_F_‐pinning regime at the conduction band, region (ii) to the unpinned regime, and region (iii) to the *E*_F_‐pinning regime at the valence band. Region (iv) corresponds to samples prepared in air. The inset in panel (a) is a schematic of the MAPbI_3_ crystal structure.

The results deduced from the above measurements are summarized in **Figure** **1**. As seen from Figure 1a,b for MAPbI_3_ films prepared under in N_2_, *Φ* increases linearly with *Φ*_sub_ in the range of 4.22–5.11 eV, corresponding to a vacuum level alignment situation (i.e., Schottky–Mott limit) at the MAPbI_3_/substrate interfaces. Accordingly, *E*_VBM_ exhibits equivalent shifts from 1.39 to 0.56 eV, yielding the same ionization energy (IE) of all samples. Decrease or increase of *Φ*_sub_ beyond the respective critical values, *Φ* ^–^ of 4.22 eV and *Φ* ^+^ of 5.11 eV, leads to pinning of *E*_F_ at 1.39 and 0.56 eV, respectively. Overall, a so‐called *Z*‐curve as a function of *Φ*_sub_ is found,^[^ ^33^ ^]^ consistent with “intrinsic” *E*_F_‐pinning at the valence and conduction band edges of the semiconductor. The fact that the slope in the unpinned regime (dashed lines between *Φ* ^–^ and *Φ* ^+^ in Figure 1) appears to be one, points toward essentially flat band conditions within the semiconductor, and thus a very low (unintentional) doping level and a corresponding long Debye length that exceeds the film thickness.^[^ ^37^, ^38^ ^]^ Note that this intrinsic pinning is different from *E*_F_‐pinning typically discussed for conventional elemental and compound semiconductors in contact with metals, where a significant gap density of states at the semiconductor interfaces pins *E*_F_ at one position within the gap.^[^ ^39^ ^]^ For a semiconductor without surface or gap states, such as many organic semiconductors, contact to a substrate with *Φ*_sub_ beyond the critical values results in interfacial integer charge transfer to establish electronic equilibrium, and *E*_F_ becomes pinned at the respective band edge at the interface.^[^ ^33^, ^40^, ^41^ ^]^ One could think that the range over which *E*_F_ is free to move should correspond to the semiconductor bandgap; here ≈1.65 eV for MAPbI_3_.^[^ ^42^, ^43^ ^]^ However, we must take into account that we measure the *E*_F_ position with UPS at the surface of a ≈500 nm thick film and not directly at the buried interface. The charge accumulated on the semiconductor side of the junction diffuses into the material,^[^ ^40^ ^]^ resulting in a narrow space charge region of a few nanometers, and concomitant band bending of a few 100 meV.^[^ ^44^, ^45^ ^]^ Consequently, the range of possible *E*_F_ movement observed at the surface of a film is narrower than the bandgap. This is already the case for a hypothetical defect‐free (gap‐state‐free) semiconductor, where the transferred charges reside in the valence or conduction bands, respectively. However, actual semiconductors feature defects, which often result in states within the energy gap. Consequently, gap states near the band edges can also accumulate charges transferred across the interface and, thus, lead to pinning already before the band edges can be reached by *E*_F_.^[^ ^46^ ^]^

In sharp contrast to the behavior of the films prepared under N_2_, MAPbI_3_ films prepared in air exhibit almost constant values of *Φ* (5.04–5.12 eV) and *E*_VBM_ (0.50–0.62 eV), regardless of *Φ*_sub_ (see Figures S2 and S3 in the Supporting Information). This implies that *E*_VBM_ at the film surface is different from that at the interface to the substrate, at least for cases where *Φ*_sub_ is below *Φ* ^+^ of 5.11 eV (as determined above). Consequently, this suggests that air‐prepared MAPbI_3_ films are significantly p‐doped and that the doping level is sufficiently high so that the Debye length is comparable to the film thickness, as, otherwise, one would expect a larger scatter of the *E*_VBM_ values as a function of *Φ*_sub_. In the following, the origin of this—apparently air induced—p‐doping is identified.

### 2.2 Energy Level Alignment of MAPbI_3_ as a Function of Oxygen/Air Exposure

**Figure 2:**
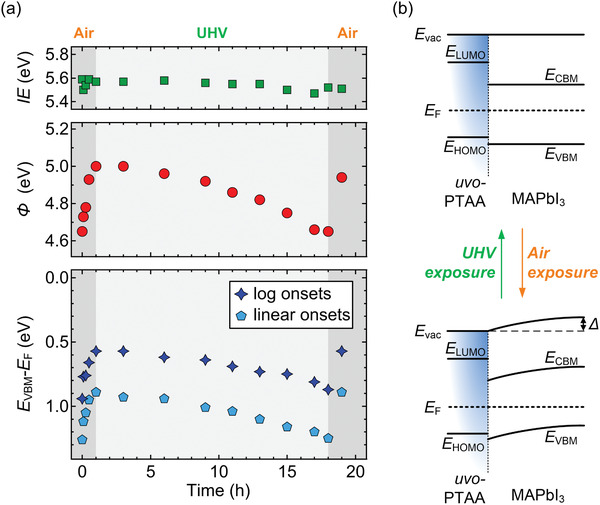
a) Summary of key electronic parameters of MAPbI_3_/uvo‐PTAA films upon successive air and UHV exposure for different periods. b) The corresponding schematic energy level diagrams of a pristine/UHV‐stored and air‐exposed uvo‐PTAA/ MAPbI_3_ sample, for the case of initial vacuum level alignment with the substrate. Complementary schematic diagrams for substrate *E*_F_‐pinned MAPbI_3_ films are shown in Figure S10 (Supporting Information). Note that the level evolution in the perovskite layer is an example and will depend on the time‐dependent oxygen distribution within the layer.

In light of the striking differences between N_2_‐ and air‐grown films, the influence of air exposure on the energy levels was explored further. First, MAPbI_3_ films, deposited under N_2_ on a UV–ozone‐treated poly[bis(4‐phenyl)(2,4,6‐trimethylphenyl)amine] (uvo‐PTAA) substrate, were exposed to ambient air for increasing time periods and at each step brought to ultrahigh vacuum (UHV) for UPS measurements (less than 1 h in UHV at each step). Representative results are summarized in **Figure** **2**; the corresponding spectra are shown in Figure S5 (Supporting Information). As discussed above, the N_2_‐prepared sample features interfacial vacuum level alignment with an initial *Φ* of 4.65 eV and an *E*_VBM_ of 0.94 eV. Upon air exposure, *Φ* gradually increases and saturates at 5.00 eV after 1 h of air exposure. Concomitantly, *E*_VBM_ shifts equivalently toward lower binding energy and saturates at 0.57 eV. The IE thus remains constant. The sample was subsequently kept in UHV (base pressure: 9.0 × 10^–10^ mbar). As shown in Figure 2, *Φ* and *E*_VBM_ values gradually revert to their initial values during storage of the sample in UHV for a period of 17 h. Finally, exposing the sample to air again leads to shifts of *Φ* and *E*_VBM_ to 5.00 and 0.57 eV, respectively. The full reversibility of the shifts excludes significant sample modification or degradation during the measurements. These findings are congruent with p‐doping of MAPbI_3_ by air exposure, and that this effect is reversible when the pressure of air is significantly reduced (UHV). At this point, diffusion of atmospheric oxygen into and out of the sample can be considered as a most likely cause.

**Figure 3:**
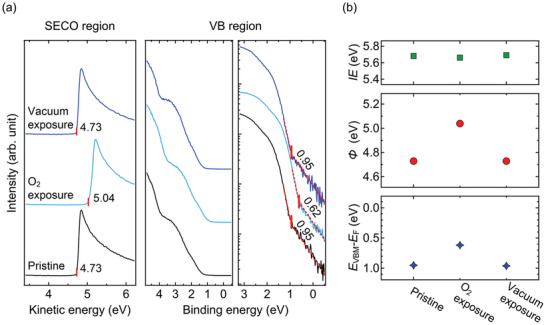
a) UPS data of a N_2_‐prepared uvo‐PTAA/MAPbI_3_ film before and after successive oxygen and UHV exposure. b) Key electronic parameters extracted from the UPS measurements.

To confirm that the predominant effect of air exposure is due to oxygen, similar experiments were performed with a N_2_‐prepared MAPbI_3_/uvo‐PTAA sample, exposed to a controlled oxygen atmosphere, and the results are summarized in **Figure** **3**. To reproduce the oxygen concentration under ambient conditions, the MAPbI_3_/uvo‐PTAA sample was exposed to pure oxygen of ≈200 mbar for 1 h in the load‐lock chamber of the UHV system used for UPS measurements (base pressure: 10^−6^ mbar). In full agreement with the trends observed for air exposure, *Φ* increased from 4.73 to 5.04 eV and *E*_VBM_ decreased correspondingly from 0.95 to 0.62 eV upon oxygen exposure. The O_2_‐exposed film was subsequently stored in UHV condition. Again, the perovskite energy levels recovered to their initial values after 23 h storage in UHV, clearly identifying oxygen as the cause for p‐doping.

The universality of the oxygen‐induced p‐doping phenomena was confirmed by performing UPS measurements on N_2_‐prepared MAPbI_3_ films on ethoxylated polyethyleneimine (PEIE) (*Φ*_sub_ of 3.8 eV) and on commercial HIL 1.3 (*Φ*_sub_ of 5.9 eV) substrates, as shown in Figures S6–S8 (Supporting Information), respectively. For MAPbI_3_ films on PEIE, after air exposure for 1.5 h, *Φ* and *E*_VBM_ gradually shift from 4.17 to 4.95 eV and from 1.41 to 0.60 eV, respectively. After storing the sample in UHV for ≈64 h, *Φ* and *E*_VBM_ are 4.27 and 1.42 eV, respectively, i.e., essentially the initial values. This also confirms the notion that the initial MAPbI_3_ film had no significant factual n‐type character, even though *E*_F_ at the surface was found close to the conduction band minimum (CBM). Instead, the material has a very low intrinsic carrier density and the Debye length exceeds the film thickness, so that an *E*_VBM_ position dictated by *E*_F_‐pinning at the interface to the substrate is observed. As expected, the energy levels of an MAPbI_3_/HIL 1.3 sample, with initial *E*_F_‐pinning close to *E*_VBM_, do not exhibit any change under both air and vacuum exposures. The observed effects, and their reversibility and long characteristic timescales, can be ascribed to the diffusion of surface‐adsorbed oxygen into the bulk of MAPbI_3_ films during oxygen/air exposure, and sublimation of oxygen molecules from the MAPbI_3_ film accompanied by out‐diffusion of oxygen from the bulk in UHV. If the p‐doping were to occur only due to oxygen on the surface, surface photovoltage would occur, which we did not observe in our experiments. Furthermore, for surface‐specific doping much faster timescales were to be expected, particularly for oxygen/air exposure (at the oxygen partial pressure used in our experiments, it takes ≈5 ns for every surface site to be hit by an oxygen molecule^[^ ^47^ ^]^). This is further supported by the few 10 s timescale observed for changing the photoluminescence yield of oxygen‐exposed MAPbBr_3_^[^ ^48^ ^]^ and a triple‐cation perovskite,^[^ ^49^ ^]^ where surface interactions dominate.

**Figure 4:**
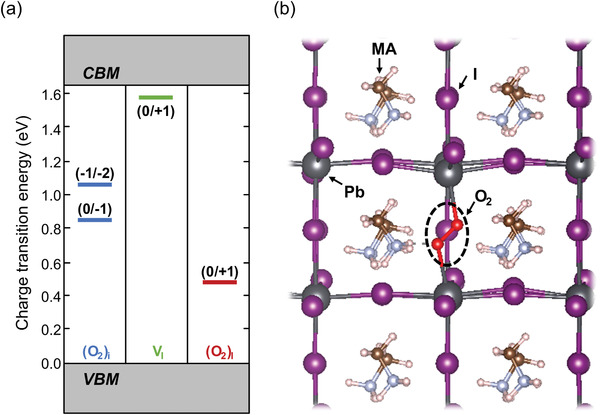
a) Calculated charge transition levels of an O_2_ interstitial [(O_2_)_i_], an I vacancy (V_I_), and a substitutional O_2_ molecule at an iodine vacancy site [(O_2_)_I_], for tetragonal MAPbI_3_. b) Minimum energy structure around the (O_2_)_I_ site, emphasized by the dashed oval. I, Pb, and O are in purple, dark gray, and red, respectively.

To obtain mechanistic insight into p‐doping of MAPbI_3_ by oxygen, DFT calculations were performed, with key results summarized in **Figure** **4**. First, an interstitial O_2_ defect was considered. However, its presence was found to only introduce midgap to slightly n‐type defect levels within the bandgap, so that it is unlikely to cause the observed p‐type behavior upon oxygen exposure. Therefore, a substitutional O_2_ molecule, occupying an I vacancy site, was considered instead. As clearly observed in Figure 4, the I vacancy energy level is rather close to the CBM and therefore exhibits an n‐type character. Introduction of an O_2_ molecule into the vacancy site results in an energy level closer to the VBM, consistent with p‐type doping. An additional mechanism—oxidation of an interstitial I to create I—O complexes (IO^−^, IO_2_^−^, IO_3_^−^)—has been considered by Meggiolaro et al.,^[^ ^50^ ^]^ and further explored by He et al.^[^ ^51^ ^]^ It was found that this too results in a lowering of the associated charge transition level, but the change itself was considerably smaller (<0.25 eV).^[^ ^50^ ^]^ We therefore do not view this as the dominant mechanism, although it certainly points to a chemical trend of oxygen counteracting the effects of I‐related defects and likely contributes further to the overall picture. The DFT results allow us, then, to explain the reversible p‐doping nature by a shift of defect energy levels brought about by oxygen exchange, as illustrated in Figure 4. In turn, the fact that O_2_ interstitials are not p‐dopants implies that native I defects are present in the MAPbI_3_ films already before oxygen exposure.

To align the above findings with the fact that our N_2_‐prepared films exhibited very low effective doping concentration (because they showed essentially flat band conditions throughout the film thickness; see Figure 1 and its discussion above), we propose that I vacancies and I interstitials are present but balance their n‐ and p‐type effects, respectively, so that overall the samples appear intrinsic. Upon oxygen exposure, O_2_ association with I vacancies changes these defects to p‐type and enhances the p‐type character of the I interstitials, with the net p‐type doping observed here being the result. As estimated in Figure S11 (Supporting Information), and its discussion therein, a net doping density of <10^10^ cm^–3^ is sufficient to move *E*_F_ ≈ 0.55 eV above the VBM. We note that this oxygen exchange picture relies on: i) effective oxygen diffusion through the solid, and ii) facile oxygen exchange between the interstitial and the ion vacancy site. The former is already known for MHPs.^[^ ^52^, ^53^ ^]^ The latter has not been tested explicitly, to the best of our knowledge. However, it is reasonable given that ion migration, including that of iodine ion itself, is well known in MHPs.^[^ ^54^, ^55^, ^56^ ^]^ Finally, we note that the timescales of oxygen diffusion through MAPbI_3_ reported here can be expected to vary for differently prepared films. Structural differences, such as type and density of native defects, crystal grain density, and film texture are likely to affect the oxygen diffusion rate.

### 2.3 Influence of Air/Oxygen Exposure on Surface States of MAPbI_3_

**Figure 5:**
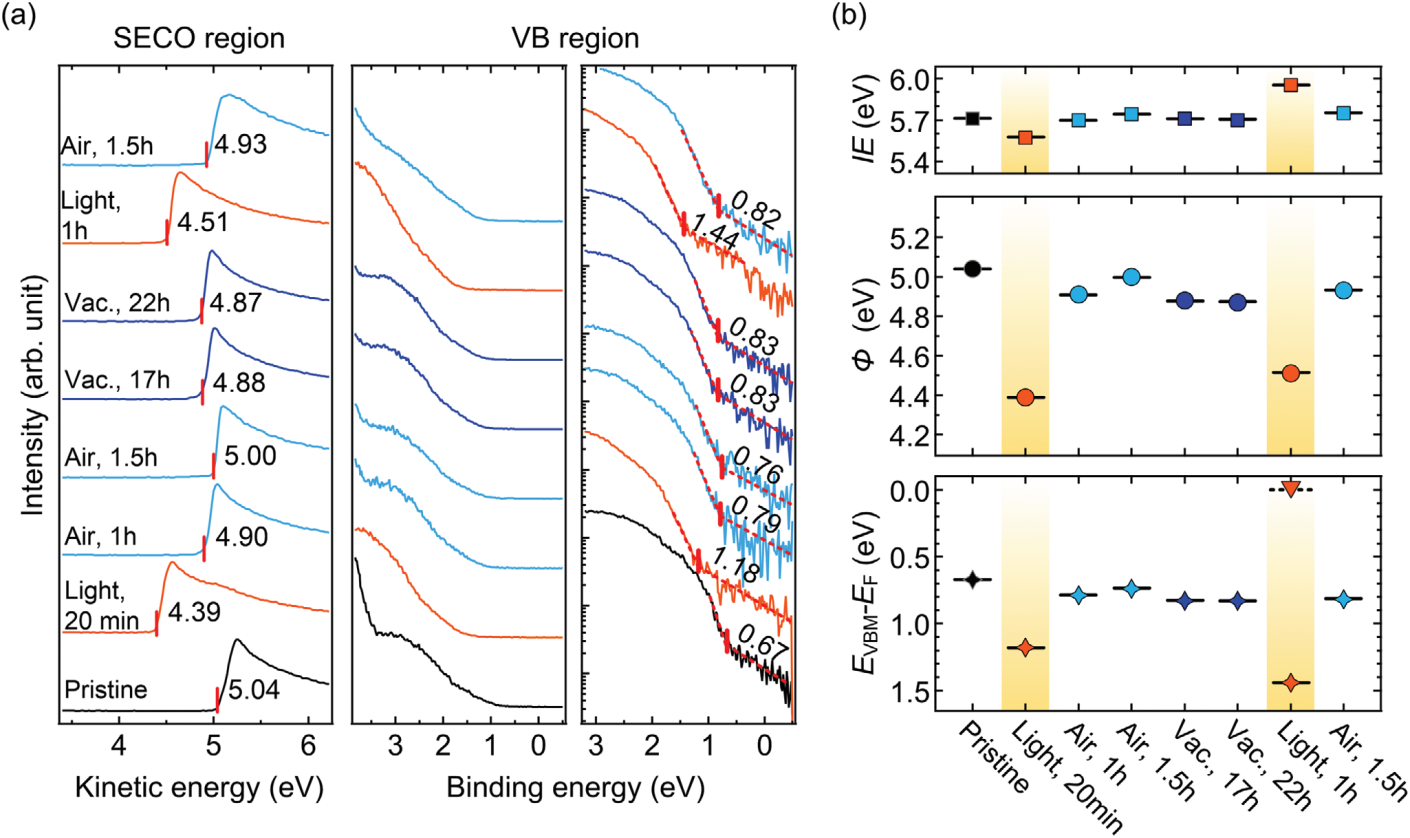
a) UPS data of MAPbI_3_/HIL1.3 films prepared under N_2_ conditions and treated consecutively by white light illumination, air exposure, and UHV storage. b) Summary of key electronic parameters extracted from the data in (a).

In addition to the oxygen‐induced bulk p‐doping effect, significant enhancement of the photoluminescence yield upon oxygen exposure was reported, in part attributed to surface defect passivation.^[^ ^48^, ^57^, ^58^, ^59^ ^]^ To consider this, an MAPbI_3_/HIL1.3 sample prepared under N_2_ conditions was exposed to white light illumination (intensity equivalent to ≈1.5 sun) for 20 min under UHV conditions, a treatment expected to promote surface I vacancies and induce the formation of metallic Pb‐related surface states.^[^ ^23^, ^60^ ^]^ With such surface states, surface photovoltage occurs upon sample illumination,^[^ ^23^, ^60^ ^]^ which was not observed for bulk O_2_‐doping (see the above). As shown in **Figure** **5**, after illumination *Φ* decreased from 5.04 to 4.39 eV and *E*_VBM_ shifted from 0.67 to 1.18 eV, indicating an induced downward surface band bending.^[^ ^23^, ^60^ ^]^ Furthermore, surface states are verified explicitly by detecting a significant density of states extending up to *E*_F_ (see Figure S12 in the Supporting Information), previously assigned to metallic Pb. Subsequently, the sample was exposed to air. As shown in Figure 5, this caused the energy levels to recover almost to their initial positions, with only a small difference of 0.12 eV despite a long storage in UHV. Furthermore, the density of surface states has vanished. Subsequently, prolonged illumination treatment in UHV for 1 h again leads to changes of *Φ* from 4.87 to 4.51 eV and *E*_VBM_ from 0.83 to 1.44 eV. This demonstrates the additional role of oxygen exposure—as a (reversible) passivation means of surface states, related to oxidation of metallic Pb.

## 3 Conclusion

We investigated the electronic properties of MAPbI_3_ films, prepared in N_2_ and in air, on substrates with a range of different work function values, and after successive exposure to oxygen and UHV, using UPS. For pristine N_2_‐prepared MAPbI_3_, we found that “intrinsic” Fermi level pinning at the band edges can be induced by choosing appropriate substrate work function values. In conjunction with the absence of notable band bending in the ≈500 nm thick MAPbI_3_ films, we concluded that the semiconductor is essentially intrinsic. In contrast, the energy levels of air‐prepared MAPbI_3_ were pinned close to the valence band maximum regardless of substrate work function, i.e., exhibited a p‐type character. From measurements with controlled air/oxygen exposure experiments we confirm that oxygen diffusion into the bulk is the cause for p‐doping, and that this effect is reversible by storing the samples in UHV for many hours, i.e., oxygen can diffuse out of the material. From DFT modeling we identified the key p‐doping contribution to stem from O_2_ substitution of iodine vacancy sites; O_2_ interstitials play no notable role for doping. Finally, oxygen exposure was also found to passivate surface Pb‐related defect states. These insights provide a comprehensive understanding of the surface and bulk energy levels of the prototypical MAPbI_3_, and show how a controlled sample environment helps to eliminate discrepancies so far observed when studying their electronic structures. This allows a much higher level of reliability for material selection during device design, as well as a reliable connection of interfacial energy levels with device functionality. It is also conceivable that other lead‐halide perovskites will undergo similar oxygen‐induced processes, but this remains to be confirmed.

## 4 Experimental and Computational Details

### Sample Preparation

Indium tin oxide (ITO) substrates were cleaned with ultrasonication in detergent, acetone, methanol, and deionized water. The wet‐cleaned ITO substrates were exposed to UV–ozone for 15 min before subsequent deposition of various organic films. HIL 1.3 (H.C. Stark GmbH) and poly(3,4‐ethylenedioxythiophene):poly(styrenesulfonate) (PEDOT:PSS, formulation AI 4083, Heraeus) were spin‐coated from aqueous dispersion onto the ITO substrates with two‐step rates at 500 rpm/5 s and 2500 rpm/25 s, respectively. For the uvo‐PTAA and PTAA w/(9,9‐bis(3‐(*N*,*N*‐dimethylamino)propyl)‐2,7‐fluorene)‐*alt*‐2,7‐(9,9‐dioctylfluorene) (PFN) films, PTAA (Sigma–Aldrich) solution was prepared at a concentration of 2 mg mL^−1^ in toluene and spin‐coated onto the ITO substrates at 6000 rpm for 30 s. The PTAA film was annealed at 100 °C for 10 min, and treated with UV–ozone for 30 s for the uvo‐PTAA film, and PFN solution (0.05 wt% in anhydrous methanol) was spin‐coated onto the PTAA film with a rate at 5000 rpm/30 s for the PTAA w/PFN film. For [6,6]‐phenyl‐C61‐butyric acid methyl ester (PCBM) films, PCBM (Sigma–Aldrich) solution was prepared at a concentration of 20 mg mL^−1^ in chlorobenzene and spin‐coated onto the ITO substrates at 4000 rpm/30 s. The coated PCBM film was annealed at 100 °C for 10 min. For the PEIE films, PEIE solution was prepared from poly(ethyleneimine) (Sigma–Aldrich) in 2‐methoxyethanol at concentrations of 0.4 and 0.2 wt%, respectively. The coated films were annealed at 110 °C for 10 min.

Thin films of MAPbI_3_ were prepared by the so‐called antisolvent treatment method,^[^ ^61^, ^62^ ^]^ which is known to result in high‐quality and low defect concentration material. The MAPbI_3_ solution was prepared with 159 mg of MAI (99%, Sigma–Aldrich), 461 mg of PbI_2_ (99.999%, Sigma–Aldrich), and 71 µL of dimethyl sulfoxide (DMSO, Sigma–Aldrich) dissolved in 0.6 mL of *N,N*‐dimethylformamide (DMF, Sigma–Aldrich).The perovskite precursor solution was spin‐coated at 4000 rpm for 30 s onto the organic films. 0.2 mL of diethyl ether was dropped onto the perovskite intermediate phase film with a delay time of 8 s after the start of the spin coating. Perovskite films were subsequently annealed at 100 °C for 3 min. The MAPbI_3_ films were fabricated in the air (relative humidity of 25%) and N_2_‐filled glove box (oxygen and water contents were less than 0.01 ppm), respectively. The perovskite films prepared in the glove box were directly transferred to the vacuum chambers for UPS measurements without air exposure.

### Photoelectron Spectroscopy

UPS measurements were conducted using a SPECS PHOIBOS 100 hemispherical analyzer system and a JEOL JPS‐9030 system. For photoelectron excitation, a monochromatized helium discharging lamp (21.22 eV) at the SPECS system and hydrogen Lyman α source (10.2 eV, Excitech)^[^ ^63^ ^]^ at the JPS‐9030 system were used, and a sample bias of −10 V was applied to obtain the secondary cutoff spectra. Binding energy shifts can be determined with ≈10 meV accuracy, and the absolute error of values given was estimated to be ≈50 meV. The base pressure of both analysis chambers was maintained below 9.0 × 10^–10^ mbar. Surface photovoltage measurements were conducted by varying the UV flux of the UPS excitation source with the monochromator settings by as much as a factor of ≈100; external visible light was blocked during these measurements. Both air and oxygen exposure experiments were conducted in a load‐rock chamber (base pressure = 1.0 × 10^–6^ mbar). For vacuum exposure, the samples were stored inside the UHV (base pressure of 9.0 × 10^–10^ mbar) chamber. Illumination treatment was conducted using a white halogen lamp (Solux, 50 W, daylight rendering spectrum, intensity equivalent of 1.5 sun).

### Computations

All DFT calculations were performed using the Vienna Ab initio Simulation Package (VASP),^[^ ^64^ ^]^ a plane‐wave basis code in which ionic cores are described by the projected augmented wave (PAW) method.^[^ ^65^ ^]^ All structures were optimized using the Perdew–Burke–Ernzerhof (PBE)^[^ ^66^ ^]^ form of the generalized‐gradient approximation, augmented by dispersion terms calculated within the Tkatchenko–Scheffler (TS) scheme.^[^ ^67^ ^]^ A 2 × 2 × 2 supercell (containing 384 atoms) of optimized tetragonal MAPbI_3_ (*a* = *b* = 8.89 Å, *c* = 12.7 Å) was used as a basis for all calculations. Defects were created by adding or removing the relevant atoms and relevant charge, and allowing the structures to relax until the forces were below 0.01 eV Å^−1^. A plane‐wave cutoff of 400 eV and a 10^–6^ eV per supercell convergence criterion for the total energy were used in all calculations. A 2 × 2 × 2 *k*‐point grid was utilized for sampling the Brillouin zone. The Heyd–Scuseria–Ernzerhof (HSE)^[^ ^68^ ^]^ short‐range hybrid functional, together with the inclusion of spin–orbit coupling (SOC) effects, was then used with the obtained geometries to produce the charge transition levels of the specified point defects. The fraction of exact exchange was tuned to α = 0.43 in order to obtain an accurate bandgap, as previously suggested by Du.^[^ ^69^ ^]^ A plane‐wave cutoff of 400 eV and a 10^–4^ eV per supercell convergence criterion for the total energy were used in all calculations. A 2 × 2 × 1 *k*‐point grid was utilized for sampling the Brillouin zone.

Charge transition levels, ε(*q*_1_/*q*_2_), were calculated for each defect using the following equations^[^ ^70^ ^]^

(1) $$\begin{array}{c} E_{\text{formation}}^{q}=E_{\text{defect}}\;-E_{\text{host}}-\underset{i}{\sum }\;n_{i}\mu_{i}+q\left(\varepsilon_{F}+E_{V}+\Delta V\right)+E_{\text{corr}} \end{array}$$

(2) $$\begin{array}{c} \varepsilon \;\left(q_{1}/q_{2}\right)=\frac{E_{\text{formation}}^{q_{1}}\left(\varepsilon_{F}=0\right)-E_{\text{formation}}^{q_{2}}\left(\varepsilon_{F}=0\right)}{q_{2}-q_{1}} \end{array}$$

where $E_{\text{formation}}^{q}$ is the energy required to introduce a defect into an otherwise pristine material, *E*_defect_ is the total energy of a cell containing a defect, *E*_host_ is the total energy of the pristine cell, *n_i_* is the number of atoms of species *i* that were added (for positive *n_i_*) or subtracted (for negative *n_i_*) from the material, μ_*i*_ is the chemical potential, *q* is the charge of the defect, ε_F_ is the Fermi energy referenced to the VBM of the pristine cell (*E*_V_) (can vary between 0 and the bandgap), Δ*V* is a term that aligns the VBMs of the defect‐containing cell and the pristine cell, and *E*_corr_ is a correction term for the electrostatic interaction between the defect and its periodic images, based on the Lany–Zunger scheme.^[^ ^71^, ^72^ ^]^

## Conflict of Interest

The authors declare no conflict of interest.

## Supporting information

Supporting Information

## Data Availability

The data that support the findings of this study are available from the corresponding author upon reasonable request.
