# Supplementary material for: Mechanism and Timescales of Reversible p‐Doping of Methylammonium Lead Triiodide by Oxygen
Source: Adv Mater. 2021 May 3;33(23):2100211. doi: 10.1002/adma.202100211 (PMC11468336; doi:10.1002/adma.202100211)
Supplement: Supplementary file 1 — Supporting Information [file ADMA-33-2100211-s001.pdf]

© 2021 Wiley-VCH GmbH

ADVANCED MATERIALS

Supporting Information

for *Adv. Mater.,* DOI: 10.1002/adma.202100211

Mechanism and Timescales of Reversible p-Doping of Methylammonium Lead Triiodide by Oxygen

*Dongguen Shin, Fengshuo Zu, Ayala V. Cohen, Yeonjin Yi, Leeor Kronik, and Norbert Koch**

[Supporting Information]

Mechanism and time-scales of reversible p-doping of methylammonium lead triiodide by oxygen

Donggeun Shin,[†,‡] *Fengshuo Zu,*[†] *Ayala V. Cohen,*[§] *Yeonjin Yi,*[⊥]

Leeor Kronik,[§] *and Norbert Koch*[*,†,‡]

[†] Institut für Physik & IRIS Adlershof, Humboldt-Universität zu Berlin, 12489 Berlin, Germany

[‡] Helmholtz-Zentrum Berlin für Materialien und Energie GmbH, 12489 Berlin, Germany

[§] Department of Materials and Interfaces, Weizmann Institute of Science, Rehovoth 76100, Israel

[⊥] Institute of Physics and Applied Physics & Van der Waals Materials Research Center, Yonsei University, Seoul 03722, Republic of Korea

Corresponding Author

*E-mail: norbert.koch@physik.hu-berlin.de (N. K.)

1. Ultraviolet photoelectron spectroscopy (UPS) spectra of $MAPbI_3$ films on the various substrates

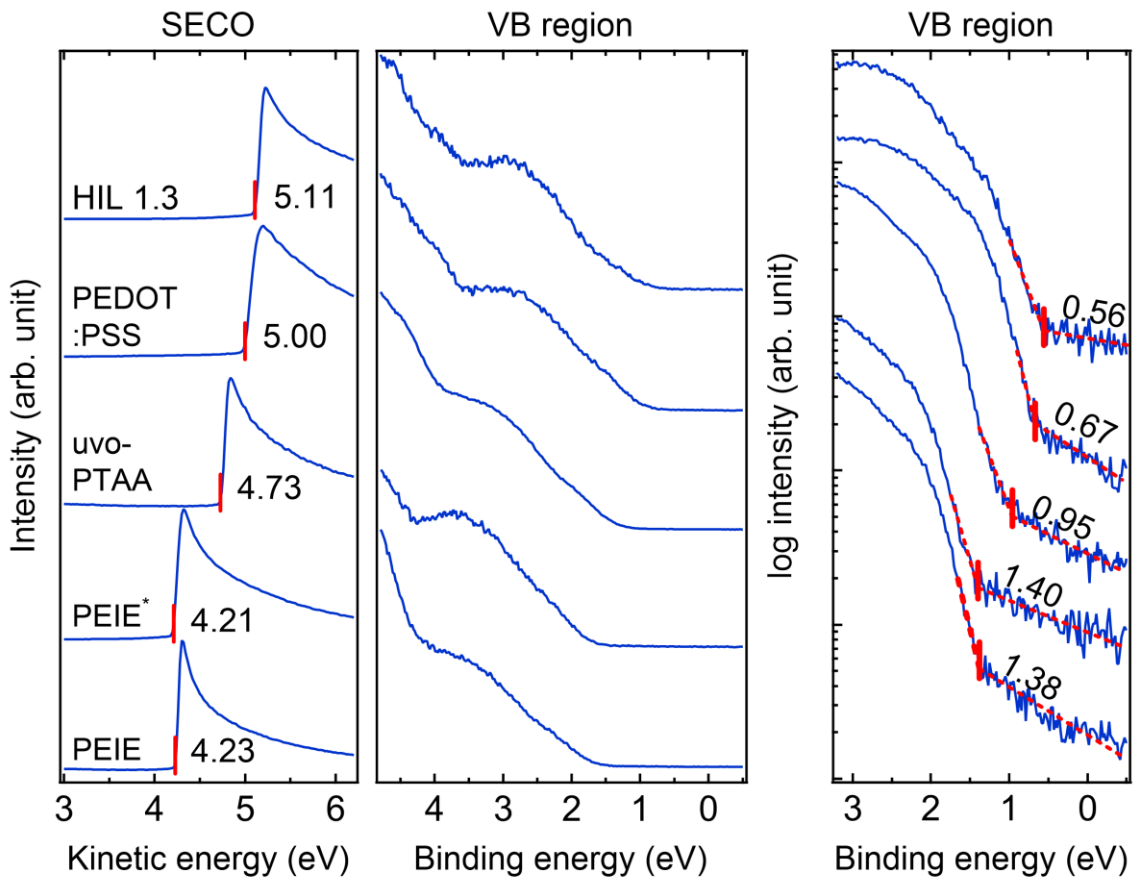

Figure S1. UPS data of N_2-prepared $MAPbI_3$ perovskite films with different substrates.

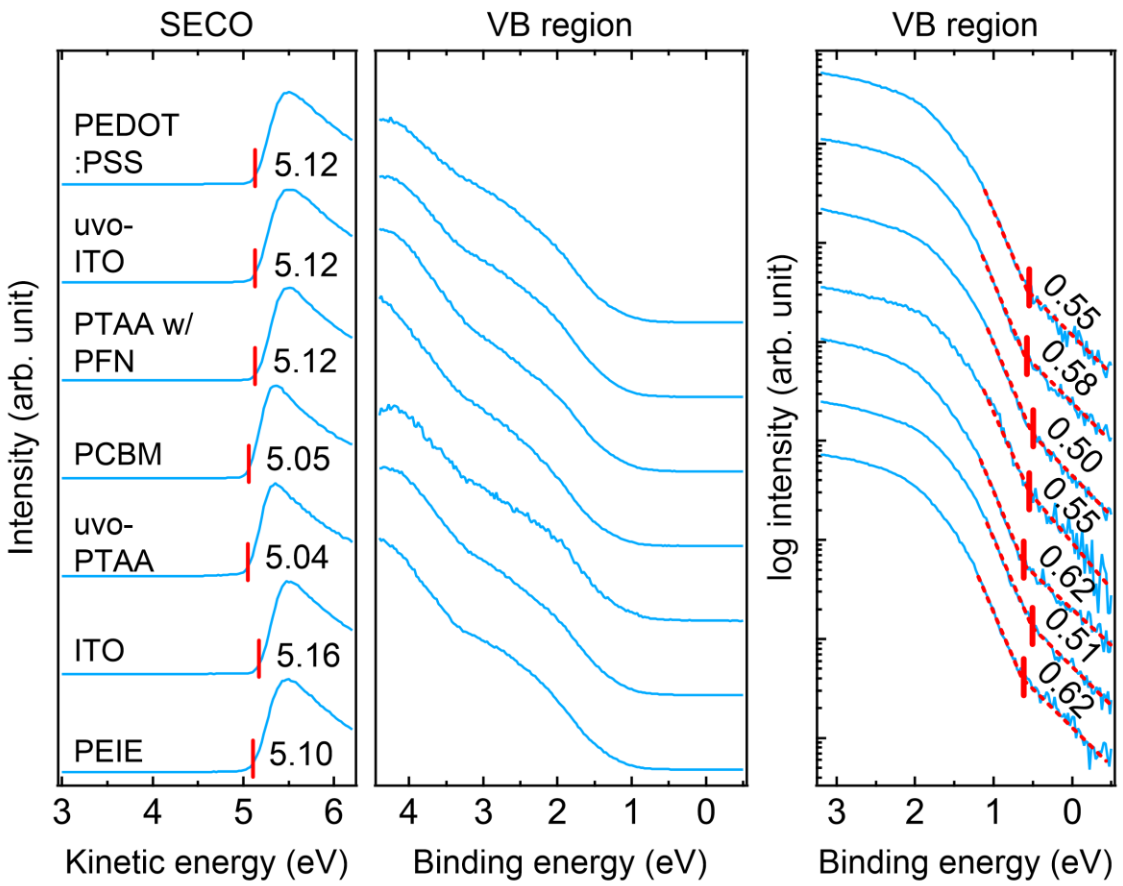

Figure S2. UPS data of air-prepared $MAPbI_3$ perovskite films deposited on different substrates.

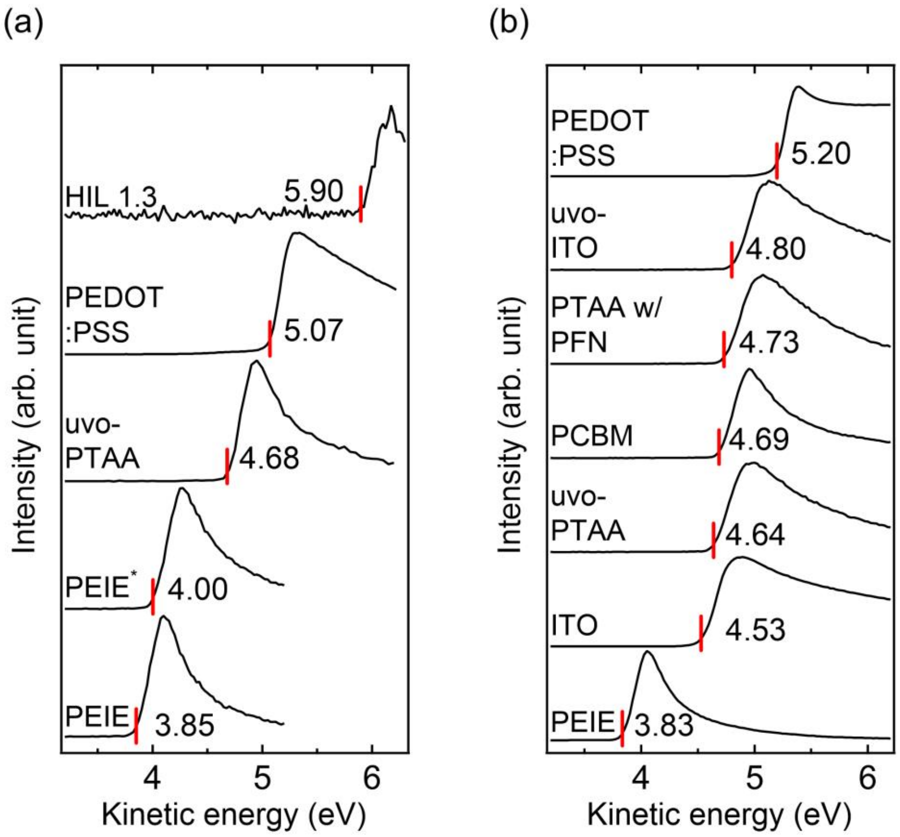

Figure S3. UPS data of the secondary electron cutoff (SECO) region of the substrates employed for (a) N_2-prepared and (b) air-prepared $MAPbI_3$ films. The corresponding sample work function values are denoted by red bars. PEIE and PEIE* denote nominally identical substrates, but with slightly different work function.

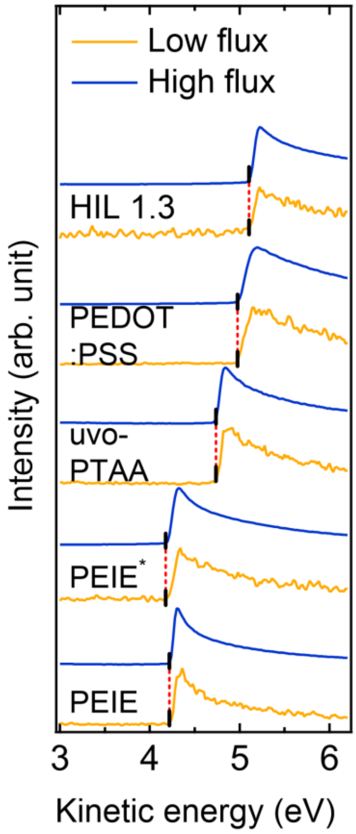

Figure S4. Secondary electron cutoff spectra of N_2-prepared $MAPbI_3$ perovskite films on various substrates under different UV fluxes (low flux is attenuated by a factor of ca. 100 compared to high flux).

2. UPS results of $MAPbI_3$ films upon air and ultrahigh vacuum (UHV) exposure

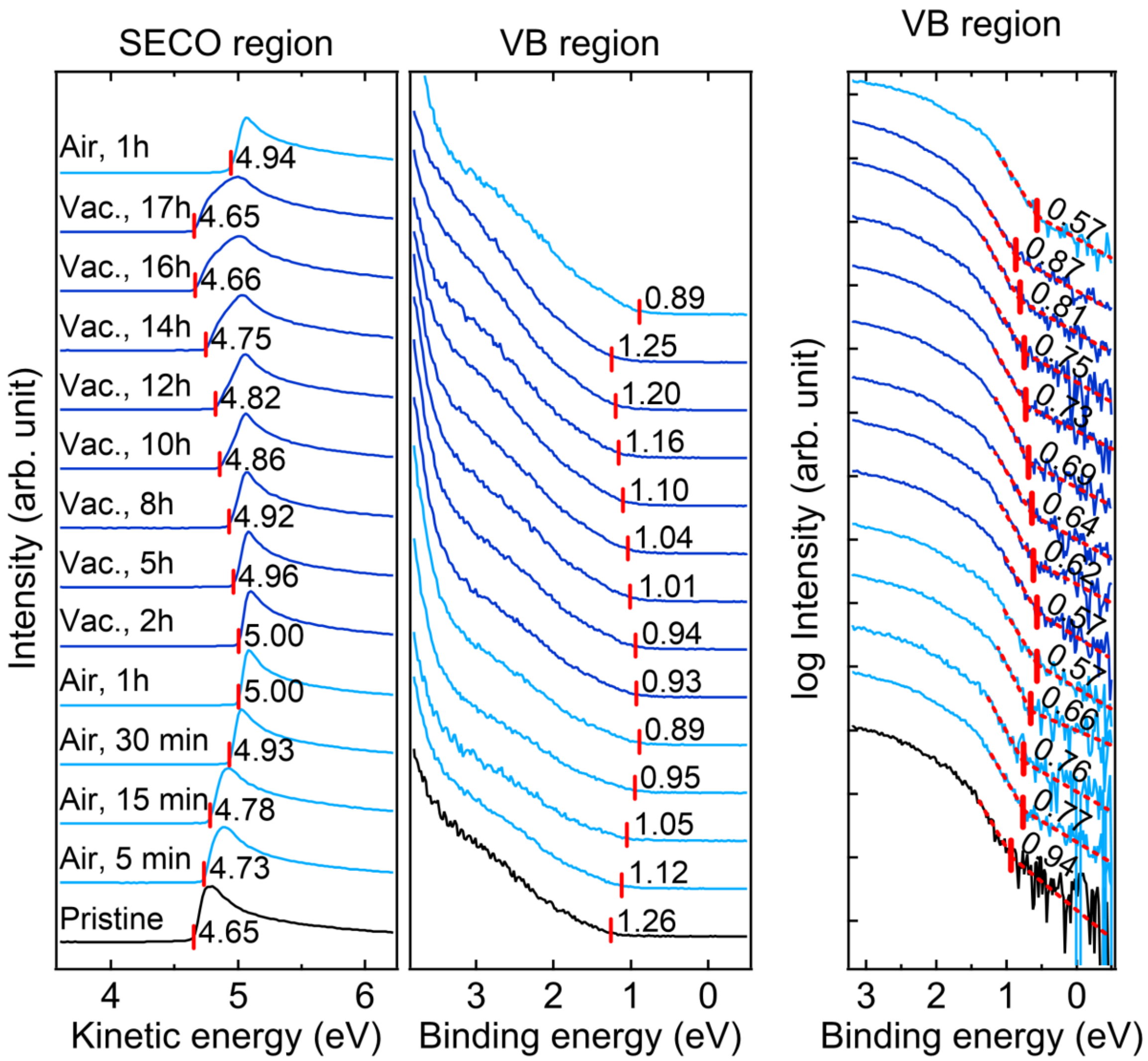

Figure S5. Time-dependent UPS data of N_2-prepared uvo-PTAA/$MAPbI_3$ films, exposed in air and UHV over multiple time intervals.

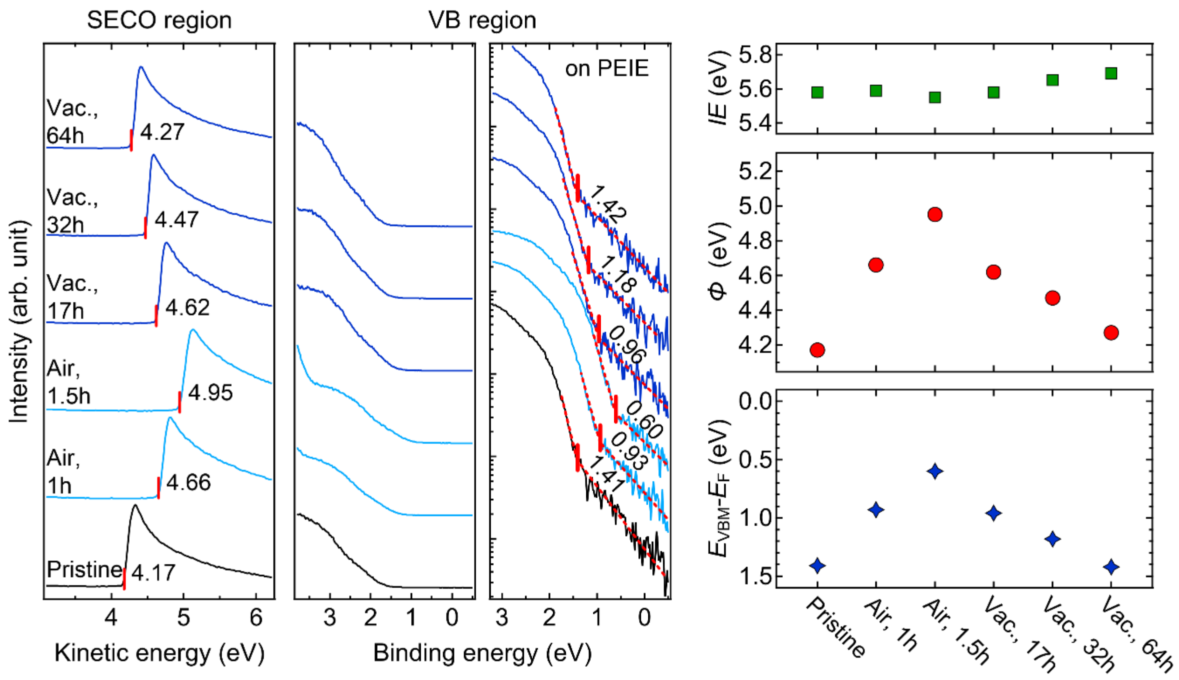

Figure S6. (a) Time-dependent UPS data of $MAPbI_3$/PEIE films, exposed to air and UHV conditions over multiple time intervals. (b) Key electronic parameters extracted from the UPS data in (a).

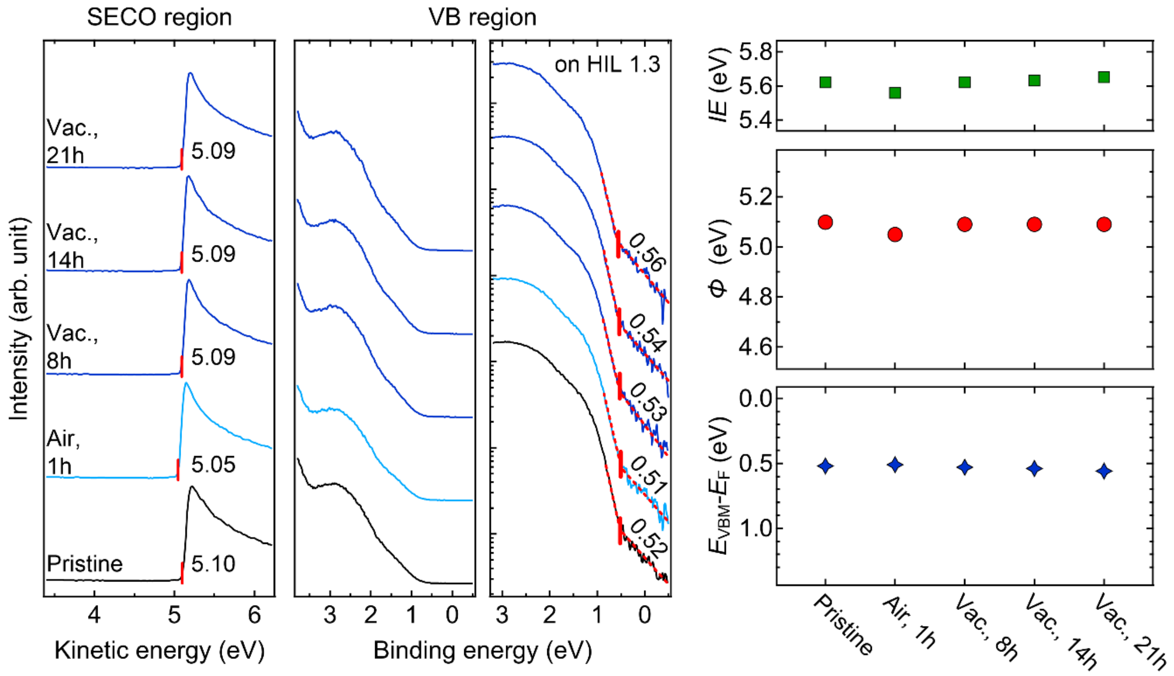

Figure S7. (a) Time-dependent UPS data of $MAPbI_3$/HIL1.3 films exposed to air and UHV conditions over multiple time intervals. (b) Key electronic parameters extracted from the UPS data in (a).

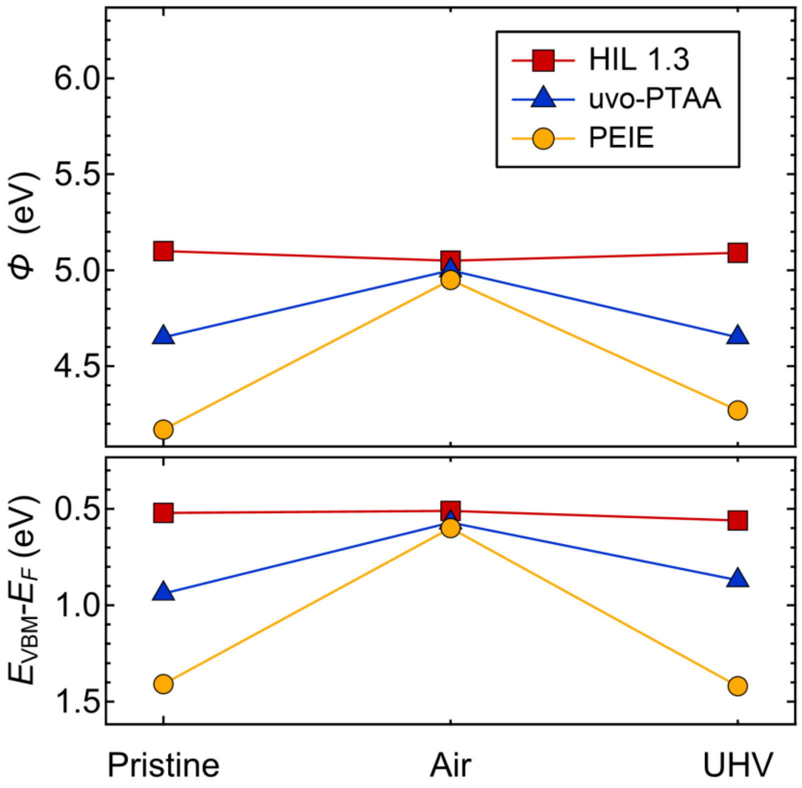

Figure S8. Shifts of work function and valence band maximum values of N_2-prepared $MAPbI_3$ films deposited on PEIE (Φ=3.85 eV), uvo-PTAA (Φ=4.68 eV), and HIL1.3 (Φ=5.90 eV) substrates, respectively, treated under successive air and UHV exposure.

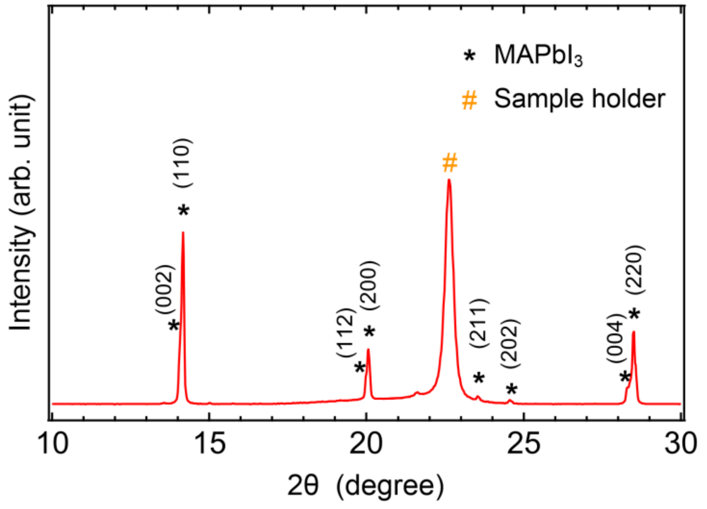

Figure S9. X-ray diffraction pattern of a $MAPbI_3$ film after air exposure for 2 hours. Reflexes of $MAPbI_3$ are indexed according to Refs. [1,2]. No indication for the presence of PbI_2 due to possible degradation is found.

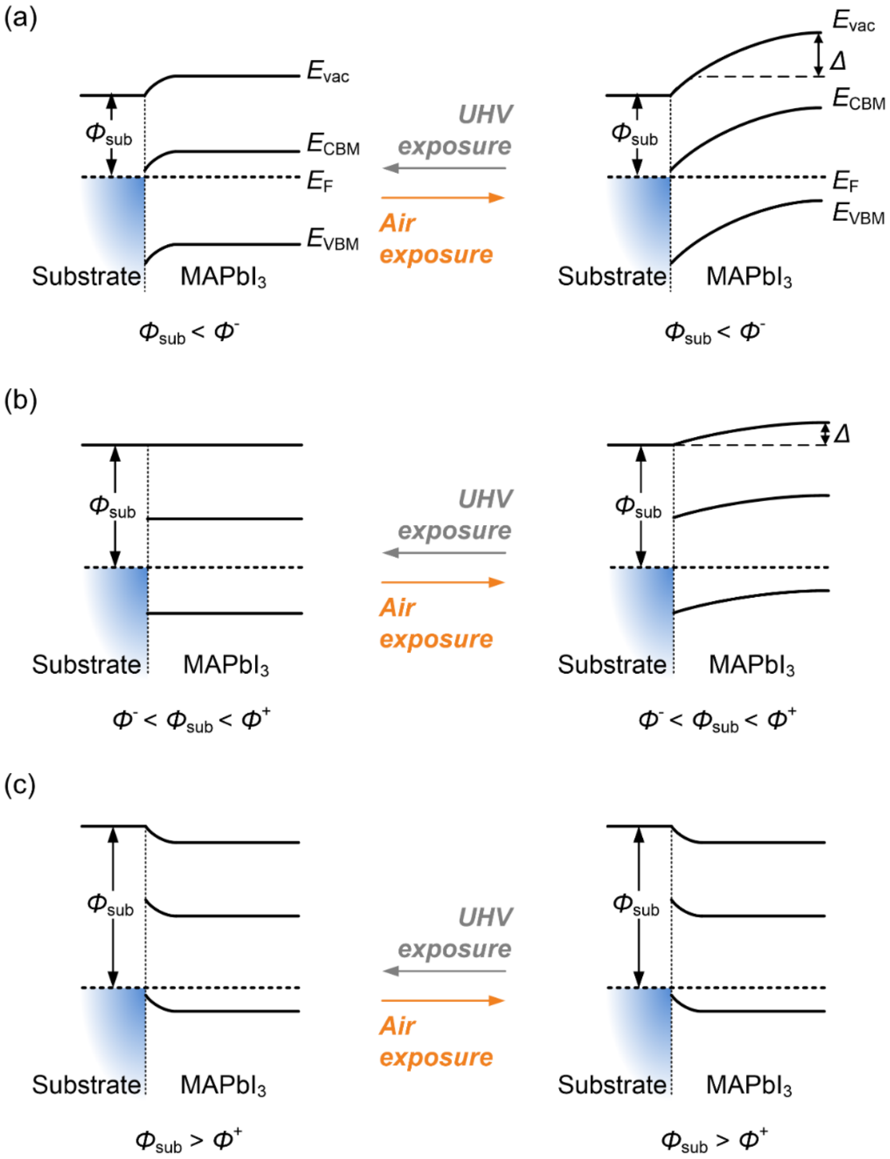

Figure S10. Schematic energy level diagrams of pristine/UHV-stored and air-exposed $MAPbI_3$ samples for the three different initial energy alignment situations with respect to the substrate. (a) E_F-pinning at E_{CBM} ($\Phi_{sub} < \Phi^-$) , (b) vacuum level aligned ($\Phi^- < \Phi_{sub} < \Phi^+$), and (c) E_F-pinning at E_{VBM} ($\Phi_{sub} > \Phi^+$), respectively.

3. Fermi level position as function of carrier concentration for $MAPbI_3$

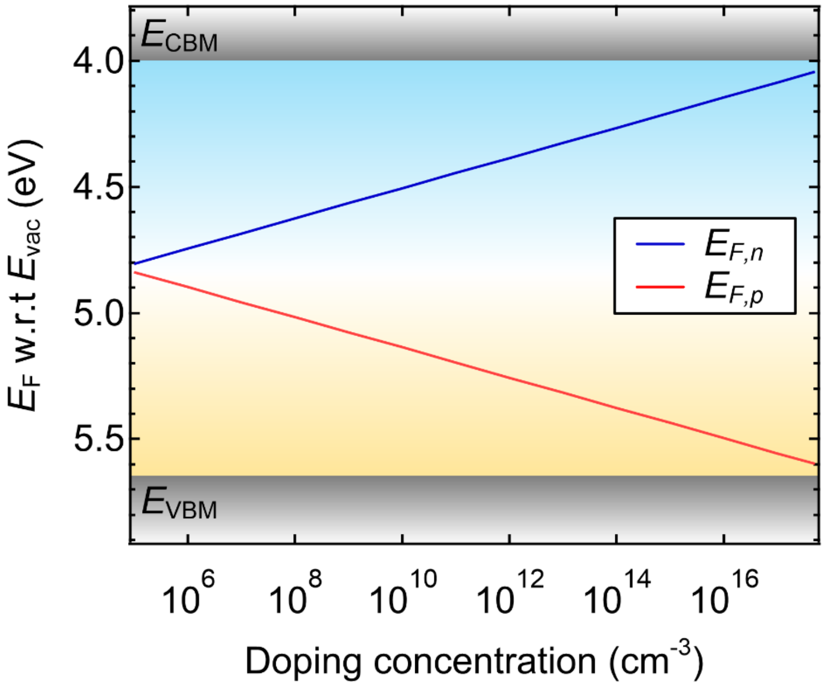

Figure S11. (a) Calculated Fermi level position as function of carrier concentrations for bulk $MAPbI_3$ (with respect to the vacuum level, E_{va})

To estimate the position of the Fermi level (E_F) as function of carrier concentration for $MAPbI_3$ films, $E_{F,n}$ for n-doping and $E_{F,p}$, for p-doping, we used the equations

$$E_{F,n} = EA + k_B T ln \frac{N_C}{n_D}$$

$$E_{F,p} = IE - k_B T ln \frac{N_V}{n_A}$$

where k_B, T, N_C, N_V, n_D, and n_A are the Boltzmann constant, temperature, effective density of states in conduction band, effective density of states in valence band, donor concentration, and acceptor concentration. N_C and N_V are given by $N_{C/V} = \frac{1}{\sqrt{2}}\left(\frac{m^*_{e/h} k_B T}{\pi \hbar}\right)^{\frac{3}{2}}$, where 2.8×10^{18} cm^{-3} for N_C and 3.7×10^{18} cm^{-3} for N_V were employed, $\hbar$, m^*_e, and m^*_h are the reduced Plank constant, electron and hole effective mass, respectively, which are assumed as $m^*_e = 0.24 m_0$, $m^*_h = 0.29 m_0$ (m_0 is the free electron mass).[3]

4. Degradation-induced metallic Pb-derived surface states

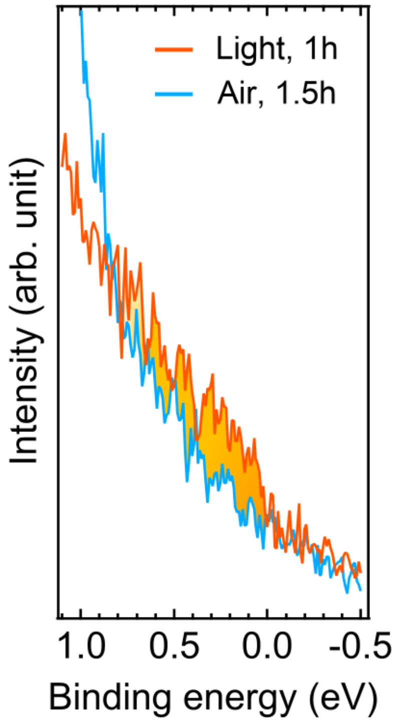

Figure S12. Magnified valence band region of $MAPbI_3$/HIL1.3 after light (for 1 hour) and subsequent air (for 1.5 hour) exposure. The orange shaded area highlights the density of states (due to metallic lead) that extends up to E_F (at zero binding energy) after light exposure, which is largely reduced after air exposure. Metallic Pb was shown to be present at the surface of light-exposed perovskite films by angle-dependent XPS,[4] and correlated with a density of states extending up to E_F in the valence region.

▪ REFERENCES

[1] T. Baikie, Y. Fang, J. M. Kadro, M. Schreyer, F. Wei, S. G. Mhaisalkar, M. Graetzel, T. J. White, *J. Mater. Chem. A* **2013**, *1*, 5628.

[2] W. Kong, Z. Ye, Z. Qi, B. Zhang, M. Wang, A. Rahimi-Iman, H. Wu, *Phys. Chem. Chem. Phys.* **2015**, *17*, 16405.

[3] G. Giorgi, J. I. Fujisawa, H. Segawa, K. Yamashita, *J. Phys. Chem. Lett.* **2013**, *4*, 4213.

[4] F. S. Zu, P. Amsalem, I. Salzmann, R. Bin Wang, M. Ralaiarisoa, S. Kowarik, S. Duhm, N. Koch, *Adv. Opt. Mater.* **2017**, *5*, 1700139.
